# The role of faith leaders in influencing health behaviour: a qualitative exploration on the views of Black African Christians in Leeds, United Kingdom

**DOI:** 10.11604/pamj.2018.30.199.15656

**Published:** 2018-07-06

**Authors:** Nii Lante Heward-Mills, Catherine Atuhaire, Chris Spoors, Ngambouk Vitalis Pemunta, Gunilla Priebe, Samuel Nambile Cumber

**Affiliations:** 1 Faculty of Health and Social Sciences, Department of Public Health-Health Promotion, Leeds Metropolitan, Leeds, UK; 2 Faculty of Medicine, Department of Nursing, Mbarara University of Science and Technology, Kampala, Uganda; 3 Research Associate, Centre for Concurrences in Colonial and Postcolonial Studies, Linnaeus University, Växjö, Sweden; 4 Section for Epidemiology and Social Medicine, Department of Public Health, Institute of Medicine (EPSO), The Sahlgrenska Academy at University of Gothenburg, Gothenburg, Sweden

**Keywords:** Faith leaders, health behavior, black Africans, health promotion

## Abstract

**Introduction:**

Black African communities in the U.K suffer from health disparities compared to the general population. This has been attributed to the lack of culturally sensitive interventions that are meaningful to them. Faith leaders are an integral part of the community and are known to have immense influence on health behaviour of congregants and community members. However, their role in health behaviour change (alcohol and tobacco use) has been largely neglected. The aim of this study is to explore the views of Black African Christians on the role of their faith leaders in their health behaviour, with particular focus on the extent of influence and mechanisms that foster this.

**Methods:**

Eight (8) semi-structured interviews were conducted with Black African Christians between the ages of 25-44, from two churches in Leeds, UK. Data were analysed using the principles of thematic analysis.

**Results:**

Findings revealed that faith leaders could play a very important role in the health behaviour of their congregants. Faith leaders are able to influence health behaviour not only on the individual level but also on a socio-cultural and environmental level. They exert such influence through several mediators including through scriptural influence, social influence and by serving as a role models. However, no single mediator has been found to be exclusively associated to health behaviour change.

**Conclusion:**

Congregants view faith leaders as having an immense influence on their health behaviour. As a community resource, faith leaders could be better positioned to organize and foster community participation in health matters. Health promoters should thus consider collaborations with faith leaders to enhance the health of their community.

## Introduction

According to Public Health England, there are health disparities between Black Africans and the general population in the England and especially in Leeds where the health of the population is poorer than the national average [1,2]. This results in poorer health, less success in accessing health-related services, and a life expectancy rate that is lower for all ages [3]. Certain ailments, like diabetes and hypertension, are up to 6 times more prevalent in Black African populations [4]. The [5] report also found out that there were substantial social and ethnic inequalities which were “entrenched and growing”. A report by the Office of the Deputy Prime Minister (OPDM) found that Black African communities are assumed to be “hard to reach” mainly because of language barriers and culture [6]. The report opined that, this notion may be due to a lack of involvement in service management and also the inability of service providers to initiate appropriate and efficient methods of engagement. Many groups are labeled “hard-to-reach” because traditional methods of researching them may not be appropriate, thus their perspectives are not properly understood [7]. Healthcare providers have faced the problem of finding practical ways of providing appropriate, culturally sensitive care for patients and families from Black African Communities [8]. Culturally sensitive interventions take into consideration the wider sociocultural values and characteristics of the intended audience [9]. Health services can thus be tailored that meet the specific needs of the population by drawing on the expertise of members of the community e.g faith leaders. Such interventions have the potential to be more meaningful and to have a higher likelihood of resulting in behavioural change [10].

Faith beliefs are important to people from African communities; they are more likely than other ethnicities to identify as belonging to a religious denomination [11]. Church-based health promotion have the potential of reducing health disparities and the church is one of the most respected and trustworthy institutions that has the potential to greatly enhance public health work [12]. It is also viewed as an agent linking African communities to the wider population [13]. Similarly, [14] reports that besides the physician or health care provider, faith leaders are another group of people with significant influence on other people's thoughts, emotions and behaviours. Spiritual leaders are able to influence health behaviour at multiple levels, from the personal to ecological levels, with “knock on” effects on the health of the community at large. This is achieved via health education and health promoting strategies [15]. Furthermore, the influence of faith leaders on health behaviour is in line with the tenets of the Ottawa Charter for Health Promotion since they are perceived as strengthening community action [15]. That is, empowering communities to participate and take control of their own affairs. As an integral member of the community, a faith leader is uniquely positioned to promote behaviour change. A review of projects involving faith-based organisations found that especially for the hard to reach populations the clergy were able to significantly facilitate behaviour change [14,16]. Other studies have also identified the importance of faith leader's influence on health behaviour [17-19]. Similarly, faith leader influence on behaviour has been attributed to Scripture-based passages that espouse the virtues of healthy living [14,20]. One scriptural passage that was a mainstay in the above mentioned studies are that “the body is the temple of God” and thus, needs to be kept holy by refraining from unhealthy behaviours. Although some studies have assumed that faith leaders have a role in influencing the health behaviour of congregants, little research exists on the extent of their influence and the mechanisms involved [21]. According to the 2011 census, 69.1% of Black Africans identify themselves as Christians in the United Kingdom (U.K) while in Leeds 73.2% identify themselves as Christians [22]. Religious denominations thus seem to be a viable avenue through which millions of people in the Black African ethnic minority can be reached.

Religious beliefs have been associated with decreased levels of alcohol and tobacco use [12,23]. Although a number of mediators and associations e.g (scriptural influence, social support, social influence and guilt) have been proposed as intermediaries between religion and health, the magnitude of effects have not been identified [20]. A central religious doctrine and a belief is that the body is the temple of God and must be treated with respect [24]. Furthermore, the Christian religion views unhealthy lifestyles such as alcohol and tobacco consumption/abuse as deviant behaviour. Such behaviours are negatively perceived. Preaching against such behaviour may serve as a motivation for adopting health promoting behaviour [20]. In comparison to the US, where the African-American faith communities have been engaged in health promotion, faith communities within Black African communities in the UK have largely been ignored in health promotion partnerships [25]. Currently, the methods of engagement include the provision of information by faith leaders to commissioners on how to engage groups, raising awareness of services and giving feedback for interventions [25]. The socio-ecological model proposes that faith leaders can shape the attitudes and beliefs of individuals as well as the social and environmental factors that influence behaviour [12]. In this way, faith leaders are able to influence behaviour change at multiple levels, with a greater likelihood of success [26]. Per the norm, most health promotion efforts, focus on behavioural change through educational activities or other individual level change strategies, neglecting the socio-cultural and environmental settings in which those behaviours exist [27]. This study sought to provide some insights on the potential influence that faith leaders could have on health behaviour and also unravel the mechanisms and factors that are associated with this phenomenon within the Black African Christian community in Leeds, U.K. Drawing on available literature, three research objectives were formulated: to explore the congregants' perceptions, on the extent of influence faith leaders could have on their health behaviour; to explore how faith leaders exert such influence (if any) on the health behaviour of congregants; to explore why congregants believe faith leaders could have influence on their health behaviour.

## Methods

**Design and population**: This exploratory phenomenological study used a qualitative data collection approach to facilitate the understanding and explanations of the meaning of occurrences and phenomena from participants' perspectives. The target population for the study was Black African Christian Community in Leeds, UK.

**Data collection tools**: Semi-structured interviews were the method of choice for this research using the interview guide. The interview guide was based on the objectives of the research. This was tailored to get answer research questions and probe responses where necessary. The responses were audio recorded. Another tool that was used was a range of probes and follow up questions that further explored participants' answers. Participants were encouraged to talk freely although occasionally prompted when they digressed. The interview began by asking general questions about religion and health. Once they seemed at ease, more specific questions relating to faith leader's influence were then asked. In the penultimate and final questions, we asked participants if they had any further contributions or questions. More often than not, this generated additional information on what had already been said thereby, enhancing the richness of data collected.

**Formulation of interview questions**: The first set of questions was focused on eliciting the extent of faith leader's influence on the health behaviour of congregants. The second line of questioning tackled the various methods that exist and are used by faith leaders to exert such influence on the health behaviour of congregants and finally the third subject area looked at why congregants were receptive to their faith leader's messages and interventions. Pilot interviews were conducted to test interview guide and assess any potential pitfalls. Prior to beginning interviews, a friendly atmosphere was created by posing ice breaking questions, smiling and maintaining good eye contact to achieve rapport.

**Recruitment and sampling**: Participants made up of members of the Black African Community in Leeds, UK were recruited through purposeful sampling using the snowballing method. They were selected because they identified as Christians and fellowshipped with a local Church in Leeds. Respondents from the age bracket (25-44) were chosen because they are most likely to engage in multiple risks taking behaviour. All participants were of African or Caribbean origin and had relocated to the UK. Their immigrant sociocultural backgrounds and belief systems identifies with the ideal sample for this research. Based on snowball purposive sampling technique, eight (8) participants were recruited from 2 pre-selected churches in Leeds, U.K. The churches selected were the Calvary International Christian Centre (Burley Road) and Redeemed Christian Church of God (Meanwood). Initially, gatekeepers were contacted informally after Sunday church service. With their help, participants for the research were informally recruited after Sunday church service and given an information sheet. They were then given a week to decide if they would participate. They were afterwards contacted to grant their availability and consent for the interviews. To minimize the prospect of respondents dropping out, not more than two referrals were used per person to facilitate a sample that is representative of characteristics and opinions of members of the black community. There was no coercion whatsoever to take part. The interviews were conducted in private rooms within the Leeds Metropolitan City Library. Research suggests that faith leaders have a significant influence on the lives of their congregants in African countries and within African communities in the diaspora [14, 20, 21]. The focus of this study was however, the congregants' perceptions of the potential influence of faith leaders on their health behaviour. The influence they wield can and is used to directly and indirectly influence the health behaviour of their congregants. The research also sought to find out the possibility of using faith leaders as an effective source of health education and promotion. The paper does not discount the fact that faith leaders are models in all aspects of social life. Faith leaders were not interviewed as only the experiences of the congregants were of interest.

**Data analysis**: Thematic analysis was used to analyse the data. However, the technique used to identify themes was the “cutting and sorting” as described by [28] in which text and quotes which seem relevant are identified, placed into piles and subsequently sorted into sub-and main themes. An inductive analytical method was used. This approach allows themes and patterns that participants identified as important to emerge naturally [29]. For the study, a constructionist paradigm was used to conduct the thematic analysis. The first step in the thematic analysis is familiarization with the data. Transcription of interviews was the starting point of familiarization. Some patterns like scriptural influence (Appendix D) were very apparent at this stage. Familiarization involved repeated reading of the data whilst actively searching for patterns. This was done with disregard for any initial thoughts or expectations. Thereafter, textual codes were generated manually from the transcribed data and “cut out”. Both latent and semantic content were coded. Coding consisted first of identifying texts and quotes that seemed important. Secondly, potentially relevant analogies, similes and metaphors were identified. This unearthed both latent and semantic codes (Appendix D). In the next step, similar textual codes were organized into sub themes and subsequently, main themes. Some codes appeared in different themes. Eighteen (18) codes were generated which were subsequently organized into five (5) main themes. The subtheme labelled “miscellaneous” did not fall under any of the five (5) themes and was thus were discarded.

**Ethical considerations**: Before approval was granted, the Local Research ethics committee of Leeds Metropolitan University ensured that the rights and welfare of the research participants were protected. Furthermore, standards for ethical conduct of research from the British Sociological Association were examined. This study allies with those standards. The main ethical consideration for the research bordered around anonymity and confidentiality of respondents. After deciding to take part in the study, participants were signposted to complete a consent form before interviews commenced. In the analysis and presentation, respondent´s identities were anonymized. In accordance with the Leeds Metropolitan University data protection policy, all information is securely preserved until all dissemination is complete. Only the research supervisor had access to it. The contact number of NHS Smoking Help, Leeds and alcoholics anonymous were provided for persons who needed further advice or help with dealing with tobacco and alcohol use. Participants were also instructed to prompt the researcher when they felt questions were inappropriate, overly intrusive or evoked undesirable emotions. In such instances, interviews were halted shortly and questions rephrased accordingly.

## Results

Five themes in regards to the potential influence of faith leaders on health behaviour of their congregants emerged from the analysis. Participants described how much influence they thought their faith leader had in their decisions to use tobacco and alcohol. Their faith leaders were able to exert such influence. Additionally, there were themes on why congregants were receptive. The total of 8 persons interviewed consisted of 5 females and 3 males. Participants were between the ages of 25-40.

### Scriptural influence

**Word of God**: All participants mentioned the bible as the major influence in their understanding of prohibitions and unhealthy behaviour. The faith-leader was seen as the conveyor of religious messages. Their role in interpreting the “word of God” was described as critical in making them a credible and influential force in positively altering healthy behaviour. *"sometimes they quote instances in the bible to support their claims and it really helps because if you are a believer in what the bible professes you do what the bible asks you to do“*(Participant 2) *”..he uses examples from the bible….think I will relate more to it on the scripture of the bible but I have also been guided by my pastor explaining the scripture more ."* (Participant 5).

**Word of God influences behaviour**: The influence of faith leaders was partly due to their use of the 'word of God' to provide advice on health behaviour. Belief in what 'the bible says' meant that, whatever message the faith-leaders relayed was seen as from a divine source. The messages were hence received with little hesitation. The phrase 'word of God' and 'bible says' were used by participants 4 and 3 respectively and exemplified congregants' attachment and belief in Scriptural passages. *" .someone who uses the word of God to buttress his point to you has more impact.. He uses the bible very well and it is only normal that you listen to someone like that"* (Participant 2) Participants unanimously agreed that using the bible, as a reference was the reason why they were more receptive to their messages.

**Body is the temple of God**: Four participants (1, 2, 5 and 8) described the body as the 'temple of God'. They added that the body should be treated with respect and dignity. This implies that believers should not indulge in unhealthy behaviour such as drinking or smoking. This was also in direct reference to scriptural teachings and eschewed how much influence the bible had in shaping congregant's thoughts on health behaviour. *"the Bible speaks about treating your body as a temple .. the bible says that you don't get fulfilled with being drunk and it tells you to protect your body"* (Participant 5).

**Severity of health behaviour/sin is based on scriptural referencing and pastoral interpretation**: The bible additionally had a direct influence on the types of messages that were delivered by the faith leaders. 6 Participants (1, 4, 5, 6, 7 and 8) felt that the only reason issues were topical or talked about by their faith leaders was because it had been referenced in the bible as a sin. They also inferred that once there was no direct mention of it in the bible, they did not regard it as an unhealthy behaviour or sin. *"So alcohol can be more referenced and can be used more than smoking, just based on that ..do not be fulfilled by drinking alcohol and getting drunkenness ..The bible is specific on drinking"* (Participant 5) *"erm….it's mostly drinking (that is topical at church) I've never heard scripture on smoking….I mean alcohol has been directly stated but not smoking"*(Participant 7) This was point of view was constantly articulated in the health enhancing behaviour participants talked about in interviews. Although participants were informed prior to interviews that the study was focused on alcohol and tobacco use, only 3 participants spoke about tobacco use as a habit that could be influenced by their faith leaders with the majority focusing on alcohol use.

**Ways and means of influencing health behaviour (alcohol and tobacco use) preaching from the pulpit to everyone**: All participants viewed their pastors as primarily disseminating health messages via preaching the 'word of God'. All participants mentioned 'preaching from the pulpit' as the most impactful and regular methods that could 'touch your heart' and had the potential of altering health behaviour. "for me, I feed more on the word *laughs*, more on the word of God so preaching from the pulpit gets to me It makes me understand. I think it is more powerful when it comes from the bible as compared to somebody telling you"(Participant 1).

**'Teaching' from the pulpit**: 5 participants (1,3,4,6,8) viewed their pastors as teachers of not only religious scriptures but also healthy lifestyle choices. Congregants considered them as having extensive knowledge in both biblical and health issues. *"some of the teachings encourage you to look after yourself teachings from the bible or certain books.. it's kind of an overlap"* (Participant 8).

**Pastors' personal relationship with congregants is integral in influencing health habits**: Six (6) participants (1,4,5,6,7,8) conceded that 'one to one' interactions were very important in shaping their health behaviour. They described how a 'personal' and 'strong relationship' they had with their pastor facilitated this.*"but if he speaks to me one to one and tells me, I feel like wow, he really cares on a personal level not just O.K everybody…..And then also seeing him personally, also him having a one to one conversation is also very helpful, then you feel like he is actually talking to you, he is not talking to everybody in the congregation"*(Participant 5) *“I think you need to form that kind of a relationship/friendship with somebody before you try and influence them(congregants) to change or to really make any changes”* (Participant 4). Within the context of this relationship, pastors were described as 'caring','loving', 'friendly' and 'welcoming;' Although there was a general consensus on preaching from the pulpit being the most widely used method, participants revealed that more personal counselling was more likely to result in behaviour change. Participants explained that through counselling, faith leaders provide guidance and advice on how to live healthy lives.

**Pastor led health promotion events**: Four (4) participants (1,5,3,8) made mention of pastor led health promotion events outside Sunday church service that are aimed at informing and altering unhealthy behaviour such as drinking and smoking. *"When you go to breakfast morning meetings, you are told that if you smoke or you drink, you are a bad person….. you are a failure ,"* (Participant 3) Participant 8 also made mention of how a decision was made by the pastor at the organisational level to publish health blogs and bulletins that tackled issues such as alcohol and tobacco use.

**Stigmatization and stereotyping**: The Christian community and particularly faith leaders stigmatized individuals who use alcohol and tobacco (participants 3, 5, 6, 7).This they noted was primarily because it was 'ungodly' and not necessarily for health reasons. Some (3 participants) revealed that indulging in alcohol or tobacco use, meant you were a 'bad person' or someone engaged in 'sinful habits' or behaviour that was 'not acceptable'. *"They will even tell you blankly that don't hang out with people who drink, smoke or if you drink, you are a bad person… you are a failure"* (Participant 3) *"There is also this perception about people that drink or smoke….if you drink or smoke, these people are the bad guys so they try to tell you not to have those kinds of friends. I am more worried about being stigmatised, being the bad person that engages in this behaviour than 'oh my health, I'm gonna smoke and I'm gonna die' so that kind of behaviour"* (Participant 5). They inferred that their pastors were deeply worried about the stigma related to such behaviour rather than the associated health implications. Additionally, participants also disclosed that because of the negative sanctions and stereotyping associated with smoking and drinking, they had to 'behave in a certain manner' as they did not want to be 'judged' by their peers and church leaders. *"Fine I might smoke or drink but then I wouldn't want to do so in front of….or I would want any of my church leaders to see"* (Participant 6).

### Reasons for faith leader's influence

**The pastor as a role model**: There was a consensus by all participants that their pastors were role models. This can be deduced from statements such as *"He is the person who does what he preaches "*. This was seen as a motivation by most participants to emulate him and not indulge in alcohol and tobacco use. According to 4 participants (1, 4, 5, 6) they 'looked up to them' while a further 5 participants (2, 3, 4, 6, 8) remarked that they were *"following their lead"* because of their exemplary lifestyles. They suggested that it was easier for them not to use alcohol or tobacco if their pastors did likewise. *"I have to follow what he does….his way of life is a very good example….seeing how he lives his life"* (Participant 2). *"We expect them to be able to nurture in the right way.. role models…as pastors we are following their lead if he is your pastor, you need somebody to look up to…you expect that from him as well* (Participant 4).

**The pastor is well respected**: A further reason given by participants for their faith leader's apparent influence over their health decisions is that they felt these leaders were the representatives of a well respectable institution and therefore commanded respect. They suggested that they had been brought up to revere and respect the authority of their pastors. In other words, they had been socialized to respect him. "I would say it's because we are told to value and respect our faith leaders more…I think it's because your parents your family respect him so much and they have put God and the pastor on a high pedestal so" (Participant 7).

**Man of God**: Four (4) participants (2,4,5,7) conceded that the pastor is a 'Man of God'. He is thus'a voice who is not just human but a voice that represents the divine'…..Participants considered him as having divine attributes and hence listening to his messages was synonymous with listening to God. *"I think he is a man of God its normal. its only right that you listen to someone versed in the word of God"* (Participant 2).

**Demi God**: Additionally, a couple of participants (3,5,6,8) elevated the status of their pastors to beyond mortal. They likened them to Demi-Gods claiming they were perceived as *' holy like', 'Earthly Jesus Christ', 'untouchable'*and sometimes worshipped by congregants. Participants noted that as a result, their instructions and messages were neither questioned nor doubted. *"We almost take them as a Mini God … ohh my pastor is not gonna be happy with me if I do this"* (Participant 5) *"people who kind of worship their pastors that's like a big influence, because these people are worshipping the pastor….like whatever they say, whatever the pastor says is done"*(Participant 3).

### Other factors involved in influencing behaviour change

**Church community and family influence**: But for participant 4, all other participants were of the view that although their pastors were a major influence, 'its' not just him….the congregation too'. Family members were equally pivotal in influencing the drinking and smoking habits of congregants. They believed that being part of this community and that through regular interaction with them, meant that they had to conform and 'portray a certain attitude' to the 'rest of the world'. They felt that being in the company of church members reinforced decisions not to use alcohol or tobacco. *"Not really, but I would say that the congregation as well. When you hang out as a church community, you see that the others are not drinking. It reinforces the view that it's not really acceptable….They are the people you interact with so they reinforce certain behaviours and decisions….yes well not just him (pastor)….the congregation too everything together I think has contributed to my decision making"* (Participant 7).

**Personal circumstances and motivations (not preaching)**: Five (5) participants (1,4,5,6,7) also acknowledged that regardless of the amount of influence the faith leader had on their health behaviour, behaviour change was dependent on the individual. They suggested that if the individual was not ready to change, pastoral influence would have very little effect. *"If that's the decision you've made and you are not willing to change it, I don't think that your pastor might be able to"* (Participant 1). *"Yes but then its inherent….. if you want to smoke you will smoke. If you want to drink you will drink, so people come to church for coming sake some come for spiritual uplifting. Some come to church because they might be having some problems and hence won't care or are more focused on themselves and their problems so they won't notice the pastor"*(Participant 6).

### Levels of influence in health behaviour change

**Direct ways of influencing behaviour**: All participants suggested that in one way or the other, their pastor had been instrumental in influencing their behaviour not to use alcohol or tobacco. This was achieved through both direct interventions and more subtlely. Some participants (1,2,5,6,) mentioned direct ways through which their pastor influenced such decisions. Participant 6 for instance, remarked that although he may drink and smoke, he wouldn't do it in the presence of his pastor. *"uuhm like,lemme just say I was going to smoke but I decided not"* (Participant 2) *"Fine I might smoke or drink but then I wouldn't want to do it in front of…..or I would want any of my church leaders to see"* (Participant 6).

**Indirect ways of influencing behaviour**: Five participants (2,3,4,7,8) cited more indirect ways that their pastors influenced their health behaviour. They discussed that because of their pastor's advice not to go out to certain social events that promoted alcohol and tobacco use, they refrained from parties, night outs and other related activities. This was perceived as limiting their exposure to alcohol and tobacco and consequently reducing the rate of consumption of these items. *"He emphasizes that we should not engage in sinful habits….. not just basically drinking and smoking but not engaging in things that will lead you to drinking and smoking like going for all those parties where you are in a place where there is alcohol and drink so you listen to music that encourages you to drink and/or smoke…. so he has a role with that"* (Participant 5).

## Discussion

Consistent themes about faith leaders and their influence on the health behaviour of congregants emerged from the data. The recurrent nature of these themes from participant's interviews also provides evidence of the potential role of faith leaders in influencing the health behaviour of Black African congregants in Leeds, UK. The present findings suggest that congregants believe that their faith leaders are a major influence in their decisions to use alcohol or tobacco. These findings are of significance to the design of future health promotion programmes aimed at the Black African minority in the UK. From the study's findings, congregants conceded that their faith leaders are seen as exerting enormous authority and credibility in their churches and communities. Previous research has shown that faith leaders play an important role in shaping the health behaviour of their congregants [21] Faith leaders have thus been credited with directly influencing their health behaviour. According to congregants, the faith leader is the one singular person with the most influence on their health behaviour. The study's findings further suggest that faith leaders are able to influence the attitudes and health behaviour of their congregants especially on alcohol consumption and somewhat on tobacco use. This has potential 'knock on' effects on surrounding communities as congregants may share and promote beliefs with family, friends and work colleagues. More subtle ways through which the faith leaders were able to influence behaviour were also cited. This included heeding his advice not to indulge in certain social activities like parties and 'clubbing' that exposed congregants to alcohol and tobacco use. The faith leader is thus an asset to the community, who addresses the health issues of church members and the community, albeit from a scriptural perspective. Community action can thus be strengthened through collaborations between faith leaders and health promoters targeting the Black community. Similar to findings in the literature sourced, one major deficiency is whether the influence of faith leaders is a causal or associated factor in changing the health behaviour of congregants [21]. Participants mentioned other factors, such as personal motivations and social influence/support as being reasons for health behaviour change. It is consequently, difficult to determine whether the influence of faith leaders is the only mediating factor in the health behaviour change of congregants. Since the functions of religious beliefs on health behaviour are difficult to measure directly, conclusions cannot be made about any given mechanism as being exclusively responsible for positive health behaviour [30].

**Reasons why faith leaders have influence on health behaviour**: Various reasons emerged on why congregants were receptive to their faith leader's influence over their health behaviour. These included Scriptural influences, faith leader as a role model, social influences, social support and fear of stigmatization. Previous studies have identified the above mentioned factors as mediating how religion impacts behaviour [20,23,30]. Findings from this study show that faith leaders exert their influence on the health behaviour of congregants through similar mediators. However, one theme that stood out and was non-existent in literature was how respect for faith leaders was culturally deeply entrenched.

**Scriptural influences**: Scriptural references by the faith leader on health behaviour were an overarching theme that was interrelated to all other themes highlighted by the research. Scriptural influence was noted as a major reason why the faith leaders were able to influence health behaviour .The faith leaders were seen as the conveyor, “a vessel” through which scriptural messages and instructions on health behaviour were transmitted to congregants. Participants believed that their faith leader's reference to scriptures urging them not to use alcohol and tobacco was one of the reasons why they are receptive to his messages. The scriptures were the primary source of information and reference for forming attitudes and health behaviour. Scriptural influence has been identified as the most common mediator through which religious persons avoid health risk such as tobacco and alcohol use [24,31]. This is perhaps due to doctrine in the bible that forbid risk taking behavior, that is the central belief that the 'body is a temple of God' and should thus be treated with respect. From the findings, it is evident that scriptural influence has an effect on all other proposed mediators. That is the authority of faith leaders; social influences and stigma are all supported by scriptures. Furthermore, analogies drawn from the bible were directly related to the severity associated with health behaviours. For instance, most participants did not think that tobacco use was of any concern, as there was no specific text referring to it in the bible. This was also manifest in the health behaviour participants chosed to discuss. Although participants were informed that the study focused on two health behaviours, a majority of respondents chose to discuss only alcohol use. The influence of the faith leaders was also reinforced by the extent of references they made to the scriptures regarding specific health behaviours. Compared to alcohol, participants revealed that tobacco use had not been categorically mentioned; evident in the relatively few number of tobacco-related sermons. To participants, the more their faith leaders talked about a specific health behaviour, the more likely they would change their attitudes and behaviour towards it.

**Culturally sensitive**: Judging from the type of issues that are deemed topical and congregants' attitudes towards them, it is very important that interventions/ health communication models that are designed for such populations are culturally sensitive. From this finding, using scriptural passages to underpin the health behaviour seems to be a method that is more meaningful to this population.

**Authority**: The research also identified authority as an important mediator in faith leader's influence. Faith denominations were viewed as respected institutions with their leaders seen as wielding enormous authority within the African community. Participants alluded to their socialization by their parents and community to respect faith leaders. Faith leaders have been known to have immense authority within the church and to use this to promote positive health behavior [32]. Some participants in the study actually described faith leader as some sort of Demi God who is so revered and influential, that they cannot be wrong and are even worshipped by some congregants.

**Role model**: Participants in this study believe the way of life of their faith leaders as being worthy of emulation and a 'gold standard' for living their own lives. Faith leaders' lifestyle was seen as an embodiment of the scriptural messages and instructions he relayed to congregants. Faith leaders' lifestyle lent more credence to his messages and interventions. Religious persons have been known to look up to persons who live a healthy life and model their health behaviour after them [30].

**Negative sanctions**: From the findings, negative sanctions by faith leaders were another reason congregants identified as motivating changes in their health behaviour. Persons who indulge in alcohol and tobacco use are stereotyped and rebuked by faith leaders. This was accompanied by feelings of guilt and embarrassment. Negative sanctions were identified as important to participants, than deteriorating health. Although this may be a mediator with negative health implications, it nonetheless provides information on how alcohol and tobacco use is perceived in Black African congregations.

**Social influence/support** The influence of faith leaders was however downplayed as the only intervening factor in shaping the behaviours of congregants. The church community and family are seen as being very instrumental to influencing the health behaviour of congregants. Although it is possible that their conceptualisation of risk and health behaviour may be informed by faith leader messages and interpretation of scriptures, findings suggested that socio-cultural backgrounds also played a role. Social groups instill a code of conduct that all congregants must adhere to, regardless of their personal preferences and habits. Being a member of this community means conforming to specific social traditions and norms. Avoiding 'unacceptable' and deviant behaviour is rewarded with approval from church member, further reinforcing it. This is possibly because social traditions and roles are clearly defined and provide members with group norms and shared beliefs that promote positive health behaviour [26]. It has similarly been confirmed that group influences from family, friends and church members are supportive of health behavior [12, 14] .This may also be due to the fear of sanctions, embarrassment and feelings of guilt by family and friends when an individual is known to indulge in alcohol and tobacco use. On the other hand, due to messages from faith leaders and one's social network, participants were less likely to spend time in bars and parties thereby limiting exposure to alcohol and tobacco [20] opine that this indirectly affects behaviour as there is less consumption.

**Ways and means**: The study also examined Black African congregants' perspectives on the potential of their faith leaders to influence their health behaviour. Through their reflection on previous instances, recurrent themes emerged from the interviews. Congregants believed that their faith leaders acted in several interlinked ways to influence their health behaviour. These strategies include: scriptural messages and instructions from the pulpit; 'One on one' counselling hinged on personal relationships with congregants; health promotion events led by faith leaders at the organisational level Similar to other findings [26] faith leaders are seen as having a lot of influence on health behaviour on multiple levels. At the individual level, faith leaders have the potential to improve the knowledge of congregants on health behaviour, and change their risk perceptions, attitudes and beliefs towards it. Faith leaders have the communication skills, knowledge of religious doctrine, and trusting relationships with congregants. They have enormous potential to carry out behaviour change interventions in their institutions and community [14]. At the level of the congregation and the community, faith leaders have the capability to instill or reinforce social norms and traditions that promote the positive health behaviours among congregants. Individual and congregation level influence on health behaviour is facilitated by the personal relationships the faith leaders have with members of their congregation. Furthermore, faith leaders are also involved at the organisational level by forming policies and interventions aimed at positively influencing health behaviour. Evidently, the role of faith leaders influences the health behaviour of congregants at multiple levels. This involves not only behavioural contexts but also social and environmental ones. The church environment [12] mentions, is a complex one, as such approaches to changes in health behaviour must address the several factors that are likely to influence the success of interventions; from the individual level to environmental and socio-cultural factors. These themes/strategies can be used to tailor future health communication and health promotion interventions targeting faith based communities within the Black African population in the UK. Faith leaders can be used to target multiple levels of behaviour change amongst congregants. Understanding the role of the faith leaders in influencing health behaviour is important for areas of collaboration to be identified by public health personnel in the quest to reduce health disparities [32].

**Limitations of the study**: The study took place in Leeds with a large international student membership; it is likely that respondents have not been in the U.K for long hence their perspectives may not be the same as Black African communities in other parts of the UK with a longer history of residence. For this reason, the perspectives of other congregants with a longer history of residency in the UK must be sought to consolidate or verify the findings of this study. Additionally, the lack of multiple coders may have resulted in the researcher's cultural and religious background being apparent in the emergent themes. The use of multiple coders would have enhanced rigour, as only objective and consistent themes, devoid of personal and theoretical biases would have emerged [33]. Finally, due to the cross sectional nature of the study, self-reported behaviour change, could not be verified over time, but only based on the responses of participants. There was also a tendency for participants to over exaggerate the influence of their faith leaders, as doing otherwise would be considered a 'sin' in Christian circles.

## Conclusion

Congregants view faith leader as having an immense influence on their health behaviour. Faith leaders exert their influence not only at the individual view faith leader, but also through social and environmental factors that can potentially lead to a higher probability of behaviour change. A faith leader is considered as a resource/asset within their community. S/he is thus, better positioned to mobilize and foster community participation in health matters. For health promotion practitioners, engaging faith leaders is important and congruent with health promotion efforts aimed at 'strengthening community action'; empowering communities to take control of their health and wellbeing as enshrined in the Ottawa Charter, 1986 [34].

**Recommendations**: Future research should seek to develop and validate these findings and determine whether the mediators proposed as intervening between faith leaders and the health behaviour of congregants are causal, associated or not related. Additionally, further research is required on how effective the faith leader is in changing the health behaviour of congregants. This can be done by directly measuring the effect and magnitude of each proposed mediator. Further research is also required on the perceptions of faith leaders' on the role they can play in influencing the health behaviour of their congregants. In this regard, faith leaders' perceptions can be compared to congregants' perceptions to identify similarities and differences. These considerations can provide deeper insight to health promoters on how interventions involving faith-based communities will be received and should be designed. The findings of this research may serve as a blueprint for health promotion professionals in formulating health communication models and strategies that are more meaningful to people in Black African communities. Faith leaders can be encouraged to deliver specific health messages, underpinned by scripture e.g. 'keeping the temple of God holy' Further research is however required to ascertain if health behaviour messages, backed by scriptural messages and delivered by faith leaders are actually effective. Further research with long term Black African immigrant residents or those in other parts of the UK might be required to validate these findings and also measure the magnitude of faith leaders´ influence on behaviour change.

### What is known about this topic

- Church-based health promotion has the potential of reducing health disparities and the church is one of the most respected and trustworthy institutions that has the potential to greatly enhance public health work;
- The influence of faith leaders on health behaviour is in line with the tenets of the Ottawa Charter for Health Promotion since they are perceived as strengthening community action;
- Faith communities within Black African communities in the UK have largely been ignored in health promotion partnerships.

### What this study adds

- By the nature of the study's design, results cannot be generalised beyond Leeds, United Kingdom. However, the study provides an insight into the role of faith leaders in influencing the health behaviour of their congregants and why, has been illuminated;
- Potentially, faith leaders can be used to reach millions of persons in the Black African Minority who are less effectively reached by traditional public health interventions and this has been shown in this study;
- The above findings can be used by Health promotion professionals to build the capacity of faith leaders to positively influence the health behaviour of their congregants and communities; However a possible drawback from the findings is that, faith leaders in a health promoting capacity may stigmatise persons who indulge in certain health behaviour, with consequent detrimental effects on mental and physical health.

## Competing interests

The authors declare no competing interests.
